# Genetic Mapping and Phenotypic Characterization of the *Drosophila* *AIF ^e04281^* Allele Reveals Mutant Clone Loss in Mosaic Eyes

**DOI:** 10.17912/micropub.biology.001854

**Published:** 2025-12-08

**Authors:** Rachel Harris, Ishaan Prasad, Sarah E. Weaver, Amer Abdulrab, Ava A. Abernathy, Amanda Ackerman, Aaron Acosta, Alexis Aguirre, Hasan Al Wakeel, Faize Alachkar, Salette Alfaro, Baylee Allen, Amar'e Anderson, Alexis Araiza, Kayla Arroyo, Kanza Arshad, Selby Artis, Merna W. Baqa, Hannah E. Bauer, Peyton L. Beduhn, Grant Boylan, Vrinda Brahmbhatt, Matthew Brock Jr., Riley Bryant, Catherine Byrne, Reyna Carreno, Alimamy Conteh, Kennedy Cox, Emily M. Daniel, Mahamad Drammeh, Briseida Espino, Nathan Farmer, Audrey Fields, Rosalinda Flores Santizo, Sema Gisele, Maria Isabella Goolsby, Efua A. Grant, Xavier Graves, Gabriella Greenlaw, Lea Guelde, Jeremiah Hall, Jeremy Hall, Zackary Harris, Spelman M. Henry, Mark Hensley, Kenyon Hurd, Malcolm Jolley, Emily A. Kalaj, Aleena Kallan, Ravneet Kaur, Chancellor Key, Makenzie Klesch, Paige Kocher, Aspen Long, Miguel Lopez, Jason J. Lucas II, Ji X. Luth, Stacey D. Marcinkowski, Guadalupe Martinez, Kitzia Martinez, Caniya McCray-Brown, Lillian McDermott, Meghan McVay, Seth Middleton, Aziz Milla, Jabari Mobley, Ryan L. Moore, Izaiah Morris, Michael Anthony Newman, Ikenna C. Nwoke, Angel Perez, Kieli D. Phillips, Mikaela Ramirez, Erika Ramos, Ali Reda, Coriss Redmond, Madeline Rentchler, Ross S Rider, Aiden Roschi, Suhana A. Rouf, Landon Scott, Krishna Shah, Connor E. Shin, Lincoln Smith, Albis Spahiu, Justin St Cloud, Imhotep Truitt, Christian Tucker, Kathryn E. Tyler, Zachary Underwood, Colin Vaughn, Lainey Vazquez, Meredith Weiss, Javaun Whitehead, Amare Williams, Faith Williams, Marissa N. Pezdek, Hemin P. Shah, Olivier Devergne, Alexandra Peister, Joyce Stamm, Jacob D. Kagey, Julie A. Merkle

**Affiliations:** 1 University of Evansville, Evansville, IN, USA; 2 University of Detroit Mercy, Detroit, MI, USA; 3 Northern Illinois University, DeKalb, IL, USA; 4 Morehouse College, Atlanta, GA, USA; 5 National Institutes of Health, Bethesda, MD, USA

## Abstract

As part of the Fly-CURE consortium, a mutant allele of *Apoptosis inducing factor* ( *AIF* ) was characterized using complementation mapping, genomic sequencing, and mosaic phenotypic analysis to investigate its role in cell growth control in *Drosophila melanogaster* . The *AIF ^e04281^* mutation dramatically reduced homozygous mutant clone size and caused morphological defects in genetically mosaic eyes. Sequencing confirmed a transposon insertion that truncates the AIF protein preceding conserved domains essential for mitochondrial function and apoptosis. The observed clone loss indicates a cell-autonomous requirement for *AIF* and supports the use of *AIF ^e04281^* as a loss-of-function background for genetic modifier screens on chromosome arm 2L.

**Figure 1. Molecular and phenotypic characterization of the AIF e04281 mutation in Drosophila melanogaster:**
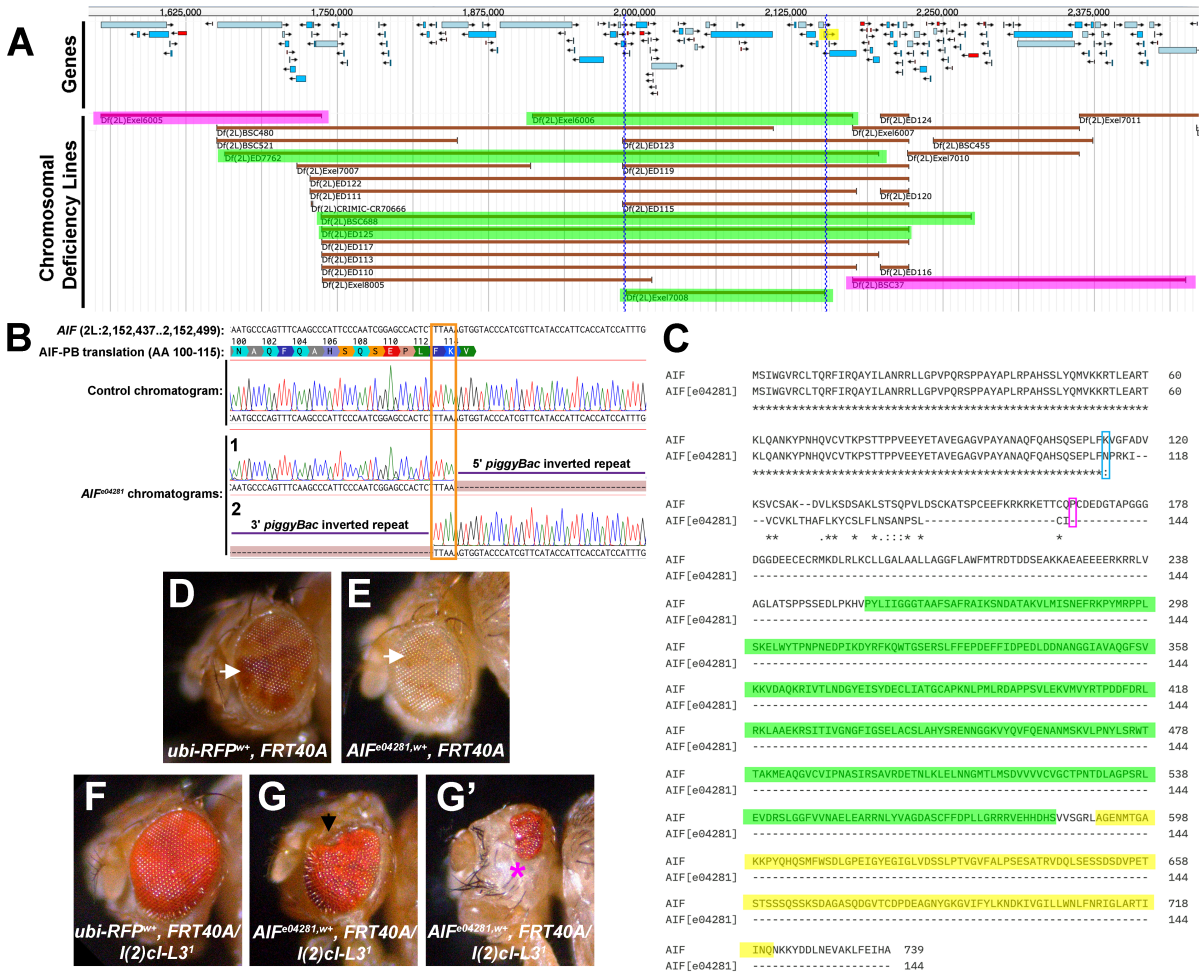
(A) Deficiency mapping localized the *AIF ^e04281^* mutation to a ~163 kb interval on chromosome arm 2L (2L:1,989,057..2,152,458, area between blue vertical lines), defined by overlapping deficiencies that failed to complement the mutant allele (green). Flanking complementing deficiencies (magenta) and *AIF* (yellow) are indicated. Adapted from FlyBase’s JBrowse ( *D. melanogaster* r6.62) (Öztürk-Çolak et al. 2024). (B) *ubi-RFP ^w⁺^ , FRT40A* (control) and *AIF ^e04281^ , FRT40A* (mutant) Sanger sequencing chromatograms aligned to the *AIF* genomic region (2L:2,152,437..2,152,499) and translation, corresponding to amino acids (AA) 100-115 of exon 2 of isoform AIF-PB. Two independent *AIF ^e04281^* chromatograms are shown, each using a different sequencing primer on opposing sides of the insertion. *AIF ^e04281^* sequence 1, generated from a primer 5’ of the insertion, includes the 5' *piggyBac* inverted repeat, while *AIF ^e04281^* sequence 2, generated from a primer 3’ of the insertion, includes the 3' *piggyBac* inverted repeat (purple lines). The TTAA insertion sequence is indicated (orange box). Adapted from Benchling DNA alignment results (Benchling [Biology Software]). (C) Clustal Omega Multiple Sequence Alignment (MSA) of wildtype and mutant AIF protein sequences revealed the location of the insertion after amino acid 114 (blue box), which introduces a frameshift and truncates the AIF protein (magenta box) (Sievers et al. 2011). The resulting mutant protein lacks conserved FAD/NAD(P)H-binding (green) and AIF_C (yellow) domains. (D-G) Mitotic recombination was induced in the developing eye using the FLP-FRT system to generate genetically mosaic tissue and assess the *AIF ^e04281^* phenotype relative to controls. Eyes are oriented with anterior to the left and dorsal at the top. (D) Control eyes (genotype: *w* - *, ey>FLP/Y; ubi-RFP ^w⁺^ , FRT40A/FRT40A* ) exhibited a higher proportion of red-pigmented tissue (white arrow; mean of 70.7% red to 29.3% white, n=40), indicating survival of homozygous *ubi-RFP ^w⁺^* clones. (E) Eyes from *AIF ^e04281^* mutants (genotype: *w-, ey>FLP/Y; AIF ^e04281,w⁺^ , FRT40A/FRT40A* ) showed a marked reduction in red-pigmented mutant tissue (white arrow; mean of 14.7% red to 85.3% white, n=40), consistent with loss or underrepresentation of mutant clones. (F-G) A homozygous cell lethal allele ( *l(2)cl-L3 ^1^* ) was used to eliminate homozygous wildtype clones. (F) Representative control eye (genotype: *w-, ey>FLP/Y; ubi-RFP ^w⁺^ , FRT40A/l(2)cl-L3 ^1^ , FRT40A* ) exhibiting normal morphology. (G-G’) Representative *AIF ^e04281^* mutants in the *l(2)cl-L3 ^1^* background (genotype: *w-, ey>FLP/Y; AIF ^e04281,w⁺^ , FRT40A/l(2)cl-L3 ^1^ , FRT40A* ) displaying smaller, irregularly shaped eyes with visible tissue defects. (G) *AIF ^e04281^* eye exhibiting ommatidial disorganization and dorsal tissue loss (black arrow) in the absence of homozygous wildtype tissue. (G’) Small *AIF ^e04281^* eye displaying strong morphological defects, including tissue overgrowth and ectopic bristles in the interocular space (magenta asterisk) when homozygous wildtype tissue is eliminated.

## Description

The Fly-CURE is a multi-institutional Course-Based Undergraduate Research Experience (CURE) that provides undergraduate students with hands-on experience mapping and characterizing novel mutants in *Drosophila melanogaster* (Merkle et al. 2023). The Fly-CURE aims to identify and study genes involved in cell growth regulation to better understand human disorders associated with abnormal cell proliferation (Neufeld and Hariharan 2002; Merkle et al. 2023; Chammout et al. 2024; Gruber et al. 2025; Patterson et al. 2025). In a previous forward genetic screen in *Drosophila* , mutant lines exhibiting defects in cell growth were identified in a background containing a mutation in *Death-associated APAF1-related killer* ( *Dark* ), a gene required for canonical apoptosis (Rodriguez et al. 1999; Mills et al. 2006; Kagey et al. 2012). When *Dark* is disrupted, cells defective in proper cell growth regulation evade apoptosis, allowing aberrant growth or proliferation phenotypes to be visualized in the developing *Drosophila* eye (Kagey et al. 2012). Following EMS mutagenesis in the *Dark* mutant background (allele *Dark ^82^* ), mitotic recombination was induced on the right arm of chromosome 2 (2R) using the FLP-FRT system to identify mutant clones with aberrant growth phenotypes (Akdemir et al. 2006; Kagey et al. 2012; Weasner et al. 2017). Students in the Fly-CURE have characterized and mapped these mutants, resulting in 17 publications (Cosenza and Kagey 2016; Bieser et al. 2018; Bieser et al. 2019; Stamm et al. 2019; Siders et al. 2021; Talley et al. 2021; Vrailas-Mortimer et al. 2021; Evans et al. 2022; Mast et al. 2022; Moore et al. 2022; Cordes et al. 2023; Nowaskie et al. 2023; Chammout et al. 2024; Johnson et al. 2024; Thomson et al. 2024; Gruber et al. 2025; Patterson et al. 2025). To extend this approach to the left arm of chromosome 2 (2L), a mutant allele of *Apoptosis inducing factor* ( *AIF* ; allele *AIF ^e04281^* ) was selected as the starting point for a new forward genetic screen (Bellen et al. 2004; Thibault et al. 2004). Because future modifier screens will rely on the *AIF ^e04281^* background to uncover mutant lines that alter eye development and cell growth, thorough molecular and phenotypic characterization of the *AIF ^e04281^* allele is essential. This study establishes that foundation and provides a critical reference point for identifying genetic enhancers and suppressors of *AIF* -dependent phenotypes.

The *AIF ^e04281^* mutation provides a foundation for studying the genetic regulation of apoptosis and tissue growth. This homozygous lethal allele results from a *piggyBac* ( *PBac* ) transposon insertion that disrupts the *AIF* gene (Häcker et al. 2003; Bellen et al. 2004; Thibault et al. 2004), the predicted *Drosophila* ortholog of mouse *AIF* and human *AIFM1* , a conserved mitochondrial flavoprotein critical for energy metabolism and induction of caspase-independent apoptosis (Susin et al. 1999; Joza et al. 2001; Joza et al. 2008; Joza et al. 2009). Studying *AIF* function in *Drosophila* can provide valuable insights into the cellular mechanisms underlying human disorders associated with *AIFM1* mutations, including Cowchock syndrome and other mitochondrial dysfunction syndromes (Rinaldi et al. 2012; Bano and Prehn 2018; Heimer et al. 2018; Nguyen et al. 2025).

To validate the genomic location of the *piggyBac* insertion in *AIF* and to establish a baseline for mapping future alleles generated in the *AIF ^e04281^* mutant background, complementation testing was conducted. Virgin females heterozygous for *AIF ^e04281^* were crossed with heterozygous males from a collection of overlapping chromosomal deficiency lines spanning chromosome 2L (Ryder et al. 2007; Cook et al. 2012). Since the *AIF* mutation and deficiency chromosomes are homozygous lethal and maintained with balancer chromosomes containing a dominant phenotypic marker that causes curly wings in adults, failure to complement results were evidenced by the absence of straight-winged progeny. Since some 2L deficiency lines did not exhibit the expected curly-wing phenotype attributed to the balancer chromosome, the collection requires re-balancing before mutants from a forward genetic modifier screen can be mapped.

Initial mapping showed that *Df(2L)BSC688* failed to complement *AIF ^e04281^* , while flanking lines *Df(2L)Exel6005* and *Df(2L)BSC37* complemented *AIF ^e04281^* , narrowing the mutation to nucleotides 1,737,249 to 2,175,620 on chromosome 2L (Table 1 and Figure 1A ). Additional deficiencies within this interval, including *Df(2L)ED125* , *Df(2L)ED7762* , and *Df(2L)Exel6006* , also failed to complement *AIF ^e04281^* (Table 1 and Figure 1A ). The smallest non-complementing deficiency, *Df(2L)Exel7008* (nucleotides 1,989,057 to 2,152,458), defined an interval containing part of the *AIF* gene (nucleotides 2,151,754 to 2,155,389), confirming the location of the mutation that causes homozygous lethality of the *AIF ^e04281^* allele (Table 1 and Figure 1A ). Complementation testing with an independent allele, *AIF ^GE14994^* , also failed to complement *AIF ^e04281^* , reinforcing that the lethal phenotype results from disruption of *AIF* and not a nearby locus (Table 1).

To validate the precise location of the *piggyBac* insertion associated with *AIF ^e04281^ ,* genomic DNA from heterozygous *AIF ^e04281^* mutant and *ubi-RFP ^w⁺^* control flies was extracted and subjected to PCR and Sanger sequencing. The insertion was reported to start at genomic position 2L:2,152,458 in exon 2 of mRNA transcripts *AIF-RB* and *AIF-RC* (Thibault et al. 2004; Öztürk-Çolak et al. 2024). Three primers were designed per student group: two to amplify the native genomic region flanking the insertion, and another targeting within the *piggyBac* insertion sequence and extending to the native *AIF* gene.

Gel electrophoresis results validated the general reported position of the insertion, and DNA sequencing from four independent sets of PCRs validated the position of the insertion at nucleotide 2,152,458 in *AIF ^e04281^* mutant DNA at the predicted *PBac{RB}* TTAA insertional target sequence ( Figure 1B, orange box) (Cary et al. 1989; Häcker et al. 2003; Thibault et al. 2004). The genomic sequence flanking this position aligned between the control and *AIF ^e04281^* sequence reads, while the remainder of the sequence aligned with *piggyBac* inverted repeats ( Figure 1B, purple lines). This insertion introduces a frameshift in exon 2 starting at amino acid 114, leading to a premature stop codon that truncates over 80% of the protein sequence ( Figure 1C ). The mutant AIF protein lacks conserved domains required for mitochondrial redox function and apoptosis, such as a mitochondrial Apoptosis-inducing factor C-terminal (AIF_C) dimerization domain and an FAD/NAD(P)H-binding (NirB) domain ( Figure 1C ) (Maté et al. 2002; Joza et al. 2008; Blum et al. 2025; Nguyen et al. 2025).

Mitotic recombination, mediated by the FLP-FRT genetic system, was used to assess the *AIF ^e04281^* phenotype in the adult *Drosophila* eye. In this system, flippase (FLP) drives mitotic recombination at flippase recognition target (FRT) sites near the centromere on chromosome 2L ( *FRT40A* ). Additionally, FLP activity is driven by enhancers of the eye-specific gene *eyeless* ( *ey>FLP* ) during development. Since the *AIF ^e04281^ piggyBac* carries a *mini* - *white* cassette ( *w ^+mC^* ), clones produced by mitotic recombination are genetically distinguished by differences in eye pigmentation: the insertion yields red pigment in *AIF* homozygous mutant and heterozygous clones, while homozygous wildtype cells lacking *w⁺* appear white. In control mosaic eyes, the average eye composition was 70.7% red tissue (homozygous or heterozygous for *ubi-RFP ^w⁺^* ) and 29.3% white tissue ( Figure 1D, n=40). In *AIF* mosaic eyes, however, red *AIF ^e04281^* mutant tissue was significantly reduced (mean=14.7%), resulting in an overabundance of homozygous wildtype tissue (mean 85.3%) ( Figure 1E, n=40). These results indicate a strong growth disadvantage or loss of *AIF* mutant cells in *AIF ^e04281^* mosaic eyes, suggesting a requirement for *AIF* in autonomous cell survival.

To investigate the role of *AIF* in cell proliferation and tissue organization, a cell lethal allele ( *l(2)cl-L3 ^1^* ) was introduced on the non-mutant chromosome to eliminate homozygous wild-type clones. After mitotic recombination, adult eyes consisted of only homozygous mutant and heterozygous cells. In *ubi-RFP ^w⁺^* controls, adult eyes exhibited a normal morphology ( Figure 1F ). However, *AIF ^e04281^* mosaic eyes often appeared misshapen and reduced in size, with variability in the presence and severity of morphological defects ( Figure 1G, G’). These results indicate that the *AIF ^e04281^* mutation leads to loss or underproliferation of mutant cells, even in the absence of wild-type competition, and that *AIF* function is critical for eye development and tissue integrity. Morphological defects included misaligned ommatidia, irregular bristle placement, and uneven interocular spacing, further supporting *AIF* ’s developmental role ( Figure 1G, G’).

In eukaryotes, loss of *AIF* function impairs mitochondrial apoptosis and may activate compensatory nuclear apoptosis pathways (Joza et al. 2008; Joza et al. 2009; Bano and Prehn 2018; Nguyen et al. 2025). The reduced presence of *AIF ^e04281^* mutant clones, along with their morphological abnormalities, reflects a cell-autonomous requirement for *AIF* in maintaining tissue viability. Interestingly, *AIF ^e04281^* mutant eyes often retained size and shape when wildtype clones were present, suggesting a compensatory proliferative response by adjacent wildtype cells. This supports a non-cell-autonomous mechanism of tissue homeostasis during eye development, wherein surrounding cells respond to clone loss (Bergmann 2025).

Because the *AIF* gene shares homology with human *AIFM1* , which regulates caspase-independent apoptosis, also called parthanatos, the *Drosophila* *AIF ^e04281^* allele provides a tractable model to study conserved mitochondrial and nuclear apoptotic pathways and their relevance to human disease (Susin et al. 1999; Fatokun et al. 2014). Disorders linked to *AIFM1* mutations include Cowchock syndrome, X-linked deafness-5, spondylometaphyseal dysplasia, and early-onset sensorimotor neuropathies (Rinaldi et al. 2012; Bano and Prehn 2018; Heimer et al. 2018; Nguyen et al. 2025). These pathologies involve impaired mitochondrial function and cell death regulation, consistent with phenotypes observed in *AIF ^e04281^* mutant flies ( Figure 1D, G,G’) (Joza et al. 2008).

Altogether, the *AIF ^e04281^* allele constitutes a lethal loss-of-function mutation in *Drosophila* and reveals critical roles for *AIF* in apoptosis, tissue homeostasis, and eye development. Its effects on clone survival and tissue morphology make it a valuable background for genetic modifier screens on chromosome 2L. Identifying enhancers or suppressors of the *AIF ^e04281^* mutant phenotype may uncover new regulators of apoptosis and growth control pathways conserved across species. These findings reinforce *Drosophila melanogaster* as a powerful model for studying the molecular mechanisms of cell growth control and their disruption in human disease.

**Table 1. Complementation analysis between *AIF ^e04281^*** **and chromosome 2L deficiency lines or an independent *AIF* allele.** Complementation testing was performed between *AIF ^e04281^* and overlapping chromosomal deficiencies on 2L. Initial results narrowed the candidate region to 2L:1,737,249..2,175,620. Additional deficiency lines within this interval also failed to complement, defining the smallest non-complementing region as 2L:1,989,057..2,152,458. A known mutant allele of *AIF* also failed to complement *AIF ^e04281^* , confirming disruption of the *AIF* gene.

**Table:** 

** Bloomington *Drosophila* Stock Center (BDSC) 2L Deficiency Kit **			
**Deficiency**	**BDSC Stock #**	**Chromosomal Region**	**Complementation Result**
*Df(2L)BSC688*	26540	2L:1,736,964..2,273,572	Fail to complement
*Df(2L)Exel6005*	7492	2L:1,555,098..1,737,249	Complement
*Df(2L)BSC37*	7144	2L:2,175,620..2,450,829	Complement
**Additional Deficiency Lines**			
**Deficiency**	**BDSC Stock #**	**Chromosomal Region**	**Complementation Result**
*Df(2L)Exel7008*	7780	2L:1,989,057..2,152,458	Fail to complement
*Df(2L)Exel6006*	8000	2L:1,911,627..2,175,599	Fail to complement
*Df(2L)ED7762*	24119	2L:1,657,408..2,197,121	Fail to complement
*Df(2L)ED125*	24120	2L:1,737,465..2,222,091	Fail to complement
**Single Gene Allele**			
**Genotype**	**BDSC Stock #**	**Gene Affected**	**Complementation Result**
* AIF ^GE14994^ /CyO *	26887	*AIF*	Fail to complement

## Reagents

*w-; PBac{w ^+mC^ =RB}AIF ^e04281^ , FRT40A/CyO* (this study; generated from RRID:BDSC_18244)

Bloomington *Drosophila* Stock Center 2L Deficiency Kit (Cook et al. 2012)

*w-, ey>Flp; FRT40A* (RRID:BDSC_5615)

*w-, ey>FLP; l(2)cl-L3 ^1^ , FRT40A/CyO* (RRID:BDSC_5622)

*w-; P{w ^+mC^ =Ubi-mRFP.nls}2L, FRT40A/CyO* (RRID:BDSC_34500)

*w ^1118^ ; Df(2L)Exel7008/CyO* (RRID:BDSC_7780)

*w ^1118^ ; Df(2L)Exel6006, P{w ^+mC^ =XP-U}Exel6006/CyO* (RRID:BDSC_8000)

*w ^1118^ ; Df(2L)ED7762, P{w ^+mW.Scer\FRT.hs3^ =3'.RS5+3.3'}ED7762/SM6a* (RRID:BDSC_24119)

*w ^1118^ ; Df(2L)ED125, P{w ^+mW.Scer\FRT.hs3^ =3'.RS5+3.3'}ED125/SM6a* (RRID:BDSC_24120)

*w-; P{w ^+mC^ =EP}AIF ^GE14994^ /CyO* (RRID:BDSC_26887)

Forward primer 1 ( *AIF* ): 5’ GTC GAT TTC AGC TCC TCT TC 3’

Reverse primer 1 ( *AIF* ): 5’ TGT CCG ACT TTA ACA CAT CC 3’

Reverse primer 2 ( *PBac* ): 5’ GTA TCG CTC TGG ACG TCA TC 3’

Forward primer 2 ( *PBac* ): 5’ CCT CGA TAT ACA GAC CGA TAA AAC AC 3’

Reverse primer 3 ( *AIF* ): 5’ TAG TCG CTT TGC AGG AAT CCA 3’
